# Intraparotid Sentinel Lymph Node Dissection for Melanoma: A Systematic Review and Meta-Analysis

**DOI:** 10.1245/s10434-024-15668-0

**Published:** 2024-07-02

**Authors:** Uriel Kfir, Ohad Ronen

**Affiliations:** 1 Azrieli Faculty of Medicine, Bar-Ilan University, Safed, Israel; 2 Department of Otolaryngology, Head and Neck Surgery, Galilee Medical Center, 221001 Nahariya, Israel

## Abstract

**Background:**

Surgical management of head and neck cutaneous melanoma (HNCM) has evolved tremendously since sentinel lymph node biopsy (SLNB) has become the prominent tool of prognosis and staging. This meta-analysis aimed to evaluate the safety and efficiency of intraparotid SLNB compared with a more extensive surgery of superficial parotidectomy (SP).

**Methods:**

The electronic database of PubMed and Scopus were searched for publications until 10 March 2022. In addition, the study included data of patients from our institution who underwent cherry-picking procedures. Pooled estimates were calculated using the random-effects model. Heterogeneity was calculated using the *I*^2^ test.

**Results:**

The pooled result regarding the rate of SLNB excision success was 97 % (95 % confidence interval [CI], 0.95–0.99; *p* < 0.0001), and the pooled probability of a positive SLNB result was 16 % (95 % CI 0.12–0.20; *p* < 0.0001). Failure of SLNB had pooled results of 4 % (95 % CI 0.02–0.06; *p* < 0.0009). For SP, no study examining N0 HNCM patients has met the authors’ inclusion criteria. Cherry-picking SLNB had temporary and permanent facial nerve paralysis relative risks (RRs) of 0.12 (95 % CI 0.06–0.27; *p* < 0.0001) and 0.46 (95 % CI 0.17–1.22;* p* < 0.0001), respectively, compared with historical data from four weighted meta-analyses of SP.

**Conclusions:**

The data from this study suggest that intraparotid SLNB performed for N0 HNCM patients is a safe and reliable procedure, with very low complication rates. Failure of the procedure did not exceed 4 %. Therefore, intraparotid SLNB may be superior to an extensive surgery such as SP and should be examined in future prospective trials.

Cutaneous melanoma constitutes 1.7% of newly diagnosed primary malignant cancers worldwide, and during the last few decades, incidences of cutaneous melanoma continue to rise, with a 3% annual increase for some populations. As of 2018, the lifetime risk for the development of melanoma in the United States is estimated to be 1 in 27 for a male and 1 in 42 for a female.^1^ Moreover, it is estimated that 20% of melanoma cases are head and neck cutaneous melanoma (HNCM), which is considered to be more aggressive, associated with increased likelihood of recurrence and diminished overall survival, than melanoma of other regions.^2^

The surgical management of HNCM regarding the necessity and extent of lymph node dissection is controversial. On the one hand, melanoma metastasizes initially to regional lymph nodes. Therefore, early excision as part of a lymph node dissection may achieve regional control. On the other hand, most patients do not have lymph node involvement, and performing such procedures carries significant morbidity.^3^ In addition, the extent of excision for achieving regional control is not well clarified. Therefore, more research together with new techniques and advances has been trying to address this issue.

Sentinel lymph node biopsy (SLNB) was first introduced for the management of HNCM in 1992 by Morton et al.^4^ It was intended as a minimally invasive procedure to determine which patients had any regional nodal disease.^3^ It became an alternative for elective lymph node dissection and radical neck dissection, which were standard management up until that time.^3^ The MSLT-1 trial followed by the DECOG-SLT and MSLT-2 clinical trials demonstrated the critical role of SLNB as a prognostic and therapeutic tool for HNCM and provided evidence for the safety and survival benefit for patients undergoing SLNB.^3,5–8^

Whereas these studies addressed a mostly surgical approach for involvement of lymph nodes in the neck, the case for parotid gland management was not well clarified. The parotid gland is a main drainage site of HNCM, with about 25% to 30% of cases demonstrating lymph node metastasis into the parotid gland.^9^ The management for patients with non-clinically apparent lymph node involvement still is highly debatable and not well addressed in the literature. Whereas paradigm-shifting SLNB studies are focused primarily on a surgical approach for lymph node involvement in the neck, the management of the parotid gland in these clinical scenarios remains poorly defined.

The two main methods for excision of parotid basin lymph nodes are dissection of the superficial lobe and sparing of its parenchyma with an intraparotid SLNB “cherry picking”-type procedure.^10^ An SLNB of the parotid glad may injure the facial nerve, and if reoperation is needed, the fibrosis might expose it to even greater risk.^11^ On the other hand, the numerous, widely distributed, and frequently occurring HNCM lymph node metastases into the parotid gland might encourage some surgeons to recommend superficial parotidectomy over SLNB.^12^ Clinical trials have been designed to evaluate these procedures for safety and outcomes, but no systematic study has compared these procedures.

This study aimed to explore the safety and efficiency of the available procedures to assist surgeons and patients in decision-making. Our hypothesis was that the intraparotid SLNB procedure does not carry increased recurrence rates or surgical complications while excising a similar number of positive lymph nodes as a superficial parotidectomy (SP).

## Methods

We followed the Preferred Reporting Items for Systematic reviews and Meta-Analyses (PRISMA) guidelines for performing a systematic review.^13^ All the studies included in this systematic review and meta-analysis were approved by the corresponding ethics committees of their institutions.

### Eligibility Criteria

The study included retrospective and prospective studies of adult HNCM patients with no evidence of lymphatic spread (cN0) and with the lymphatic drainage pattern of their primary tumor mapped to the parotid area who underwent either SLNB or a parotidectomy. The studies had to report the number of patients who had a successful identification and excision of sentinel lymph nodes in the parotid area. The exclusion criteria ruled out studies that referred to a very specific population or location, previous oncologic treatment of skin cancer in the head and neck region, or clinical evidence of known metastatic disease.

### Data Sources and Search Strategy

The electronic database of PubMed and Scopus were searched with the exclusions “NOT case reports” and “NOT reviews.” The search was conducted on 3 October 2022. The search strategy included terms and synonyms for “melanoma AND parotid AND sentinel,” and “melanoma AND parotidectomy.” The search also targeted papers on HNCM patients who were part of larger studies.

### Study Selection

The titles and abstracts were screened for the eligibility criteria by both authors. Those with the potential for relevance were reviewed thoroughly by those authors. Publications that were borderline for the inclusion criteria were discussed between the authors. Overlapping cohorts were identified using text, institution, and time periods. For these, the publication with the largest population of interest for this review was included.

### Data Extraction

The data were extracted with careful evaluation of relevancy and eligibility by two authors working independently. Characteristics of the population sought by the study were N0 melanoma with mapping to the parotid area of patients who underwent SLNB of the parotid or an SP. Data regarding study population and characteristics, Breslow depth, and results of the procedures in question were obtained. The main variables of interest were the success rate of SLNB defined as successful excision of at least one node per patient, positive and negative biopsy results, and morbidity and recurrence rates. In addition, data on complications of SP performed after SLNB were collected. False-negative was defined as regional disease recurrence in the parotid or neck after a negative pathologic result.

No study involving N0 melanoma patients who underwent an immediate SP was found according to the inclusion criteria. Therefore, we compared the data collected from the SLNB studies with published metanalysis studies on SP.

We performed a systematic search based on our inclusion criteria and performed analysis of the results using the statistical program R. Pooled estimates were calculated using the random-effects model, which provided probability of events based on the studies included. Heterogeneity was calculated with the *I*^2^ test.

We also included our institution’s retrospective HNCM patients, who were mapped for intraparotid lymphatic drainage in this meta-analysis. This part of the study was approved by the institutional review board (0166-22-NHR).

## Results

**Fig. 1:**
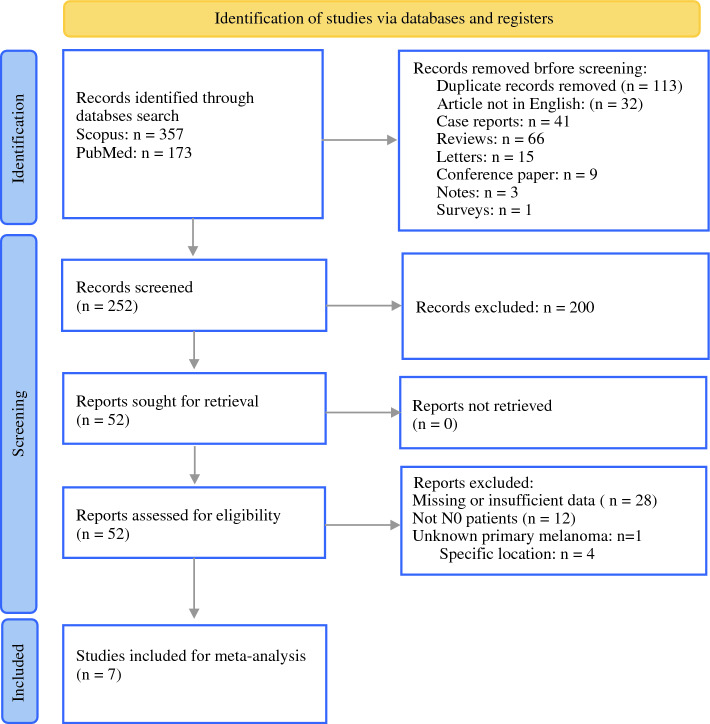
PRISMA flow chart of systematic study selection for SLNB of the parotid and superficial parotidectomy in HNCM patients. SLNB, sentinel lymph node biopsy; HNCM, head and neck cutaneous melanoma

The literature search yielded 530 papers, 7 of which met the inclusion criteria (Fig. 1). Table 1 presents the key study design elements of all the included articles.^11,14–19^ The reports, published between 1999 and 2019, encompassed a total of 344 patients, with cohorts ranging in size from 17 to 105 patients. A follow-up period was unavailable for one study because it was reported for the entire study population and not specifically for our included patients. Six of seven articles were retrospective, and the remaining article was prospective. Together with the included articles, six patients from our institution were analyzed in the meta-analysis. These patients underwent SLNB for N0 melanoma of the HNCM.

**Table 1:** Main characteristics of the studies included in the review

Author	Year	Drainage mapped to parotid/total participants	Male *n* (%)	Age (years)	Breslow (mm)	Study design	Follow-up (months)	Preoperative technetium 99 with intraoperative gamma probe localization and blue dye	Preoperative technetium 99 with intraoperative gamma probe localization alone	Intraoperative blue dye alone	Mean SLNs excised
Samra et al.^11^	2012	55/58	38 (65 %)	Mean 59.7	Mean 2.26	Retrospective	Mean 29	55	0	0	N/A
Doting et al.^7^	2006	17/36	N/A	N/A	>1	Prospective	N/A	17	0	0	N/A
Hanks et al.^8^	2019	105/356	N/A	N/A	Mean 2.23	Retrospective	N/A	N/A	N/A	N/A	2.94
Loree et al.^9^^\^	2006	28/75	25 (89 %)	Mean 66	Mean 2.3 Median 1.9	Retrospective	Mean 30.6 Median 18.4	1	27	0	1.321
Ollila et. Al.^10^	1999	39	35 (89 %)	Mean 56	Median 1.6	Retrospective	Median 33.2	39	0	0	2.3
Wells et al.^12^	1999	28	23 (82 %)	Median 60	Median 2.19	Retrospective	Median 12	22	0	6	N/A
Picon et al.^13^	2006	72	55 (76 %)	Median 62	Median 2	Retrospective	Median 26	60	12	0	2.5
Present series	_	6	2 (30 %)	Mean 60	Median 2.75 Mean 2.53	Retrospective	Mean 40.3	6	0	0	2.33

SLN, sentinel lymph node; NA,

### SLNB in the Parotid Area

**Fig. 2:**
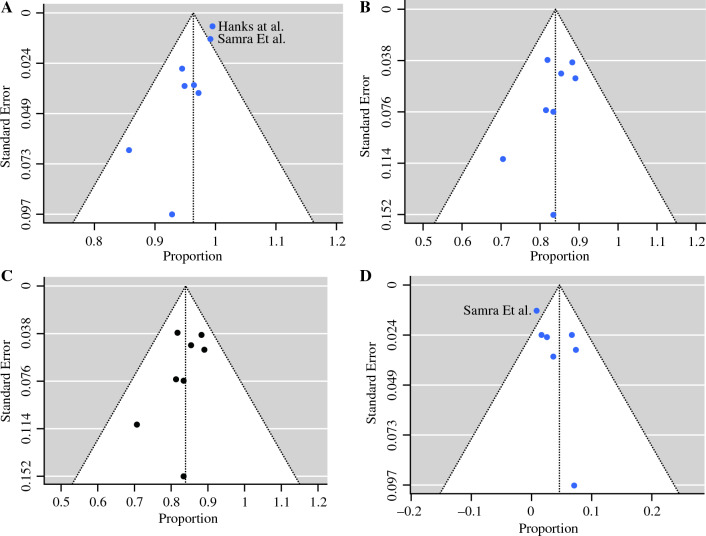
Funnel plots of SLNB analysis. Each line represents a separate study. **A** Succesful SLNB excision. **B** Positive SLNB result. **C** Temporary facial nerve injury. **D** Regional recurrent disease for pN0 SLNB patients. SLNB, sentinel lymph node biopsy

**Fig. 3:**
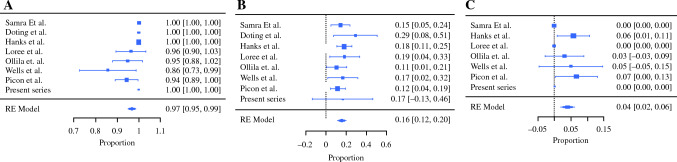
Forrest plots of SLNB analysis. Each dashed line represents a pooled proportion. **A** Successful SLNB. **B** Positive SLNB results. **C** Regional recurrent disease for pN0 SLNB patients. SLNB, sentinel lymph node biopsy

All eight studies used preoperative technetium with intraoperative gamma probe localization to map the lymphatic drainage pattern from the primary tumor. Seven of the eight studies also used blue dye intraoperatively. The pooled results of SLNB showed a successful excision rate of 97 % (95 % CI 0.95–0.99; *p* < 0.0001) in all eight studies (350 patients). Five of the eight studies reported a mean number of sentinel lymph nodes successfully excised, ranging from 2.3 to 2.94 lymph nodes in four studies, with a mean of 1.3 lymph nodes reported in the remaining study (Figs. 2a and 3a).

The pooled probability of a positive SLNB result was 16 % (95 % CI 0.12–0.20; *p* < 0.0001) for all successful SLNB excisions, (reported in all 8 studies, total of 340 patients; Figs. 2b and 3b). All but 4 of 54 patients with positive results underwent subsequent SP with or without neck dissection. For 40 of these patients, only one complication, a temporary facial nerve injury, was reported. The four patients who did not have a subsequent SP were free of disease for the mentioned follow-up period of the studies (3 patients in the Picon et al. ^19^ study with a median follow-up period of 26 months for entire cohort; 1 patient in the current study with a follow-up period of 16 months).

### Complications and Comparison with Superficial Parotidectomies

In the absence of data on the complications experienced by the N0 HNCM patients who underwent immediate SP, we compared data from four weighted meta-analyses of SP. The complications reported were temporary and permanent facial nerve damage and Frey's syndrome, which were absent with the SLNB procedure.

**Fig. 4:**
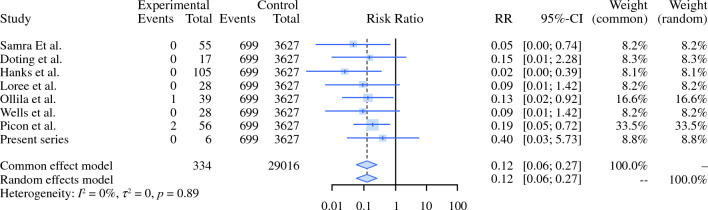
Forest plot comparing SLNB with historic series of SP for temporary facial nerve injury. SLNB, sentinel lymph node biopsy; SP, superficial parotidectomy

Table 2 presents the key study elements for the articles we analyzed.^20–23^ All the articles in the SLNB series reported the incidence of facial nerve injury, with a relative risk for temporary facial nerve damage of 0.124 (95 % CI 0.056–0.273; *p* < 0.0001). Three events for a total of 334 patients were reported by the eight articles (Fig. 4). Seven of the eight studies reported that no other complications occurred, and the remaining article did not report whether other complications occurred (see the funnel plot in Fig. 2c).

**Table 2:** Articles included in the analysis for SP morbidity.

Author	Year	Underlying disease	Procedures compared	Temporary facial nerve injury events	Permanent facial nerve injury events	Frey's syndrome events
Forest et al.^21^	2022	Benign parotid tumors	Superficial parotidectomy vs extracapsular dissection	N/A	93/2520	306/2024
Xie et al.^23^	2015	Benign parotid tumors	Superficial parotidectomy vs extracapsular dissection	178/759	17/809	135/709
Chiesa-Estomba et al.^20^	2021	Benign or malignant tumors	N/A	(IFMN) 66/288	(IFMN) 20/288	N/A
Mashrah et al..^22^	2021	Benign parotid tumors	Five different procedures^a^	455/2580	52/1913	358/2608

SP, superficial parotidectomy; N/A,

^a^Enucleation, extracapsular dissection, partial superficial parotidectomy, superficial parotidectomy, total parotidectomy.

**Fig. 5:**
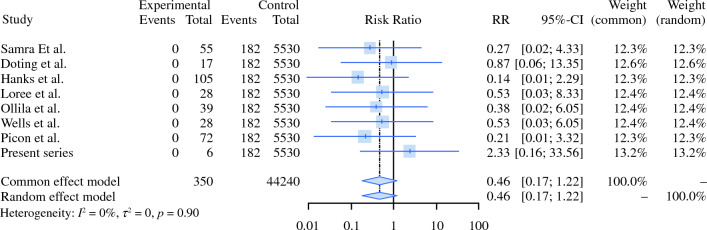
Forrest plot comparing SLNB with a historic series of SP for permanaent facial nerve injuries. SLNB, sentinel lymph node biopsy; SP, superficial parotidectomy

No events of permanent facial nerve damage occurred in the SLNB group versus 182 (3.3 %) of 5530 events, in the SP group. The RR for permanent facial nerve damage in the SLNB compared with SP was 0.46 (95 % CI 0.17–1.22; *p* < 0.0001); Fig. 5).

### Disease Recurrence After SLNB

Disease recurrence to any region had a pooled proportion of 8.3 %, with 24 recurrent events in a total 290 patients (reported in 6 of the 8 articles). Failure of the procedure was defined as the experience of a negative SLNB and a regional recurrence. Of 274 patients reported, 11 (in 7 of the 8 articles) had a regional recurrence during the follow-up period, representing a pooled proportion of 4 % (95 % CI 0.02–0.06; *p* < 0.0009; Figs. 2d and 3d).

Of 156 patients, 2 died of the disease in the follow-up period reported by five of the eight studies (pooled estimate of 0; 95 % CI 0.000–0.017). The one patient, who died 11 months after diagnosis because of metastases to the brain, had a negative result for four excised sentinel lymph nodes.^18^ The other patient was from our series. He had a negative SLNB result for the two nodes excised from the parotid, experienced systemic recurrent disease 39 months after surgery, and died 54 months after SLNB.

## Discussion

For diagnosis and treatment of melanoma, SLNB is the recommended procedure, offering less morbidity and similar overall survival.^7^ This systematic review and meta-analysis provided data demonstrating the safety of intraparotid SLNB compared with SP. Our results indicated that SLNB is a safe and reliable procedure, with weighted summary estimates of false-negative rates of 2.2 % and a weighted morbidity rate of 0 %, which strengthens its role for HNCM patients with intraparotid lymph nodes. Nonetheless, the follow-up periods vary between studies (Table 1), with a minimum median of 12 months and maximum mean of 40 months. Longer follow-up periods are needed for better determination of survival rates.

Our search did not yield any article with sufficient data on SP for cN0 HNCM. However, data collected from other meta-analyses of SP showed much greater morbidity than we found during SLNB in our series. The relative risk of temporary and permanent facial nerve injuries for SLNB were 0.12 and 0.46, respectively. These data should be interpreted with caution because patients and their comorbidities varied. Certainly, to evaluate the true morbidity of SP for cN0 melanoma patients, further studies are needed.

A study by O’Brien et al.^24^ published in 1994 evaluated parotidectomies of 107 patients, with elective SP and neck dissections for 82 patients, 12 of whom had cervical lymph node metastasis. Therefore, this study could not be included in our meta-analysis. However, its results may help surgeons in deciding on the initial operation. In 82 elective SPs, 33 patients had facial nerve injury. The report does not mention how many had a permanent or transient injury, so it is not mentioned whether facial nerve monitoring was used. In addition, only 2 of the 82 patients had a positive result on histology for occult parotid gland metastases.

Our analysis of seven articles and our own case series of six patients is rather small. Therefore, conclusions are limited and should be interpreted with caution. Nevertheless, we recommend that management of cN0 HNCM with lymphatic drainage to intraparotid lymph nodes should initially be addressed with the cherry-picking SLNB technique without excision of the superficial parotid. As our meta-analysis and review demonstrated, it is a rather safe and accurate procedure, with a similar regional control rate while carrying a lower morbidity of facial nerve injury and Frey’s syndrome. In our collected data, four patients had no subsequent SP after a positive SLNB result and were free of disease for the duration of the follow-up period. However, further studies evaluating the necessity of subsequent surgical intervention for patients with positive SLNB results who had longer follow-up periods should be performed.

It also is important to note that some of the articles in our review made a distinction between “parotid parenchymal lymph nodes” and “peri-parotid lymph nodes.” For our meta-analysis, both were considered as parotid-area lymph nodes. It is possible that such a distinction might be important for the management of specific cases. However, as shown by our pooled data, the high rates of nodes excised and the low morbidity rates suggest that in both cases, SLNB is safe and efficient in detecting occult disease in the parotid of patients with HNCM.

## Study limitations

This review had several limitations. Most of the articles reviewed were reporting retrospective studies, and some of the studies had a small sample. Also, the four articles presented in the historic meta-analysis did not involve melanoma patients inclusively. Furthermore, follow-up periods for SLNB studies vary and are limited. This limitation makes it difficult to assess the true mortality rates. In addition, information in the literature regarding SP for cN0 melanoma patients is lacking, and a direct comparison with intraparotid SLNB could not be performed.

In summary, to the best of our knowledge, this is the first systematic review and meta-analysis to evaluate the safety and accuracy of intraparotid SLNB for cN0 HNCM patients. The study found that cherry-picking SLNB is a safe and accurate procedure for the treatment of this population. Moreover, we found that the alternative surgical option of SP has greater morbidity than cherry-picking SLNB in the parotid basin.
